# Effects of Energy and Dietary Fiber on the Breast Development in Gilt

**DOI:** 10.3389/fvets.2022.830392

**Published:** 2022-03-10

**Authors:** Shengyu Xu, Lianchao Tang, Haitao Xu, Yi Yang, Meng Cao, Sirun Chen, Xuemei Jiang, Jian Li, Yan Lin, Lianqiang Che, Zhengfeng Fang, Bin Feng, Yong Zhuo, Jianping Wang, De Wu

**Affiliations:** ^1^Animal Nutrition Institute, Sichuan Agricultural University, Chengdu, China; ^2^Key Laboratory of Animal Disease-Resistant Nutrition, Ministry of Education, Chengdu, China; ^3^Key Laboratory of Animal Disease-Resistant Nutrition and Feed, Ministry of Agriculture and Rural Affairs, Chengdu, China; ^4^Key Laboratory of Animal Disease-Resistant Nutrition, Chengdu, China; ^5^Animal Husbandry Development Center of Changyi City, Changyi, China

**Keywords:** gilt, mammary glands, energy, fiber, proteomics

## Abstract

To study the effects of energy and dietary fiber on breast development in gilts and its possible mechanisms, 32 gilts (Landrace × Yorkshire) were randomly allocated into a 2 × 2 factorial design to receive a diet with low or high energy [LE: 33.37 MJ/d digestible energy (DE); HE: 41.87 MJ/d DE] and low or high fiber (LF: 0.3 kg/d dietary fiber, HF: 0.6 kg/d dietary fiber). The weight of breast tissue was recorded. The mammary glands were collected for further analyses. The high energy intake increased the relative weight of breast tissue (*p* < 0.05) and the content of breast fat (*p* < 0.05). At the same time, the oil red staining of breast slices also showed an increase in breast fat content in high-energy treatment. High energy intake increased the DNA concentration in breast tissues (*p* < 0.05). In addition, high energy intake increased the concentration of triglycerides, free fatty acids, and total cholesterol in the blood of gilts (*p* < 0.05), and the supplementation of high fiber tended to reduce free fatty acids, total cholesterol, and estradiol (*p* < 0.1). Proteomic analysis suggested that there were notable differences in the cytoskeleton, intracellular non–membrane-bounded organelle, apoptosis, receptor activity, and endopeptidase inhibitor activity in molecular function between the energy and fiber effects (*p* < 0.05). High fiber intake also decreased the mRNA expression of *5-HT7, Bax*, and *caspase-3* in the breast tissue of gilts (*p* < 0.05), which further confirmed the importance of fiber in regulating breast development in gilt. Our results indicate that increasing gilt energy intake improved breast weight and fat deposition and increased breast cell apoptosis. Increased fiber intake reduced breast fat deposition and breast cell apoptosis at high energy intake in gilts. These results provide a potential strategy for dietary intervention against high energy intake in gilts and even in humans.

## Introduction

Mammary gland secretes milk to provide nutrition and immune barrier for newborns (1–3). Therefore, the development of the mammary gland is crucial for the next generations. For the sows, the development of the mammary glands can be roughly divided into three periods, namely, early puberty (approximately 3 months), late pregnancy (about 3 months), and lactation (4–6). Studies have shown that pregnancy is the most prosperous period of mammary gland development; also, there were many studies that focused on this period (7–10). Weldon found that when feeding pregnant sows with different energy levels, the total amount of mammary DNA in high-energy treatment was 30% higher than that in normal-energy treatment; however, the total amount of mammary gland RNA and total mammary gland protein were significantly higher in normal energy treatment than the high-energy treatment (7). In lactating sows fed different energy-level diets, it was found that the percentage of protein, fat, and DNA in the mammary glands of sows increased with the increasing of gross energy, which suggests that gross energy intake affects the composition of mammary gland tissue (11). However, studies have also shown that high energy intake during pregnancy has side effects on mammary gland development and subsequent lactation (12–14). However, there are few studies on mammary gland development in puberty. Restricted feed intake by 20% from 90 days of age until puberty decreased growing gilts' mammary mass of parenchymal and extraparenchymal tissues (15). Restricted feeding from 90 days old to puberty significantly reduced mammary gland development during puberty in gilts. When the feeding amount is limited to 33% (compared with ad libitum feeding), the mammary gland weight reduced by 36.8%, and mammary DNA and RNA decreased (16).

In the production of replacement gilt, increasing the energy supply of the diet would make the replacement gilts grow quickly to meet the breeding standard as soon as possible (17, 18), but it will cause excessive obesity of the gilts, which often leads to mammary gland fat deposition and dysplasia, affecting milk production; furthermore, it has adverse effects on the lactation function in the later period (10). However, the question is how to balance the high-energy needs of gilts for rapid development and the adverse effects of excessive energy on mammary gland development. This question still needs further exploration.

Dietary fiber is called “the seventh nutrient” and is widely used in sow diets. Adding an appropriate amount of dietary fiber reduced constipation during pregnancy (19), mitigated sow delivery stress (20), and improved sow reproductivity (21), while reducing the cost of feed (22). Peffer and Rozeboom (23) used high-fiber diets (35% ground sunflower hulls) to slow the growth of growing gilts and alternated with phases of normal growth. On day 110 of gestation, they found that the treatment gilts had fewer mammary parenchyma than control gilts. However, the treatment gilts consumed more feed and tended to wean heavier litters during lactation. This showed that although the mammary gland development before lactation was decreased, high-fiber diets before puberty improved the lactation performance of gilts (23).

Therefore, we hypothesized that supplementation fiber at high-energy diet could improve mammary gland development in gilts. The current study was undertaken to investigate the effects of energy or fiber on the mammary gland development in gilts, as well as its potential mechanisms at the proteomics level. It is supposed to provide some proteomics mechanistic insights into the application of energy and fiber to a gilt diet to improve mammary gland development at the puberty periods.

## Materials and Methods

### Ethics Approval

The present experiment was conducted at the Research Farm of Animal Nutrition Institute, Sichuan Agricultural University, Ya'an, China. All experimental procedures followed the current law regarding animal protection and were approved by the Guide for the Care and Use of Laboratory Animals prepared by the Animal Care and Use Committee of Sichuan Agricultural University (Permit No. SICAU2015034).

### Animals and Diets

A total of 32 gilts (Landrace × Yorkshire, 91.70 ± 3.70 kg, 161.5 ± 0.5 days old) were used in a 2 × 2 factorial design trial: (1) LELF = LE (33.17 MJ/d digestible energy [DE]) + LF (0.3 kg/d dietary fiber, basil diet); (2) LEHF = LE (33.17 MJ/d DE) + HF (0.6 kg/d dietary fiber); (3) HELF = HE (41.87 MJ/d DE) + LF (0.3 kg/d dietary fiber); and (4) HEHF = HE (41.87 MJ/d DE) + HF (0.6 kg/d dietary fiber). Each treatment was repeated eight times, and there was one gilt for each repeat. The dose of energy and fiber chosen for this study was in line with that used in our previous study (24). The diet was formulated to meet nutritional requirements recommended by the National Research Council 2012 (NRC 2012). Based on a corn-soybean meal, the basal diet (LELF) included 13.82 MJ/kg DE, 14% of crude protein, 0.76% Lys, 0.55% calcium, 0.52% phosphorus, total crude fiber 3.3% (calculated value), and total dietary fiber 12.42% (analyzed value). The basal dietary provided 298 g fiber and 33.17 MJ DE per day (feed 2.40 kg/gilt per day). For high-energy and high-fiber treatments, soybean oil (240 g/d) or dietary fiber (300 g/d, composed of inulin and cellulose 1:4) was added, respectively, to provide additional energy and fiber based on the basal diet. Thus, the daily nutrient intake was identical between the different treatments, except for energy and fiber.

### Sample Collection

The day of gilt with the first estrus was recorded (indicated as a standing reflex in response to the manual application of pressure to the gilt's back). All gilts were slaughtered on the morning of day 19 of the fourth estrus cycle. Before slaughter, 10 mL blood sample was collected by an acute jugular puncture to obtain the plasma and serum. Briefly, plasma samples were collected using heparin as an anticoagulant (25). For serum sample collection, the blood samples were allowed to clot for 30 min before centrifugation for 15 min at 3,000 *g* (26). The plasma and serum were stored at −20°C for further analysis. The third mammary gland tissue from the left side was collected from each gilt and rapidly frozen in liquid nitrogen and then stored at −80°C (10). The fourth mammary gland tissue from the left side was fixed in 4% paraformaldehyde. The right-side mammary gland tissue was used to record the weight and sagittal area.

### Plasma Analyses

The concentrations of triglycerides (TGs), nonesterified fatty acid (NEFA), total cholesterol (TC), low-density lipoprotein cholesterol (LDL-C), and high-density lipoprotein cholesterol (HDL-C) in plasma were detected by commercial kits according to the manufacturer's instructions (Nanjing Jiancheng Bioengineering Institute, Nanjing, Jiangsu, China; catalog no. A110-1-1, A042-2-1, A111-2-1, A113-2-1, A112-2-1, respectively). Optical density (OD) values were determined at 520, 440, 546, and 510 nm by a MuLtisKan MK3-Thermo Labsystems microplate reader (Thermo Labsystems, CA, USA). Serum estradiol, prolactin, testosterone, and progesterone were measured using enzyme-linked immunosorbent assay kits (R&D Systems Inc., Minneapolis, MN, USA; catalog no. KGE014, DPRL00, KGE010, DYC5415-2, respectively). OD values were determined at 450 nm by a MuLtisKan MK3-Thermo Labsystems microplate reader (Thermo Labsystems). Minimal detection limits for TG, NEFA, TC, LDL-C, HDL-C, estradiol, prolactin, testosterone, and progesterone were 0.02 mmol/L, 10 μmol/L, 0.1 mmol/L, 0.01 mmol/L, 0.01 mmol/L, 1 pg/mL, 10 mIU/L, 1 pg/L, and 10 pg/mL.

### Ether Extract and DNA Concentration of Mammary Gland

Mammary gland tissues were analyzed for ether extract (EE) corroding to the method 920.39 of AOAC, 2006 (27). DNA concentrations of mammary glands were detected by commercial kits according to the manufacturer's instructions (TIANGEN, Beijing, China; catalog no. DP304). Briefly, it contained the steps of tissue sample digestion, lysis, and column purification. One microliter of the extracted DNA stock solution was pipetted; a Nanodrop ND-1000 spectrophotometer (ThermoFisher, CA, USA) was used to detect the purity and concentration of the analyzed samples, and the DNA content per milligram of tissue was calculated.

### Oil Red O Staining of Mammary Gland

Fixed mammary gland tissues were frozen, and 5-μm sections were cut using a Leica microtome (Leica, Solms, Germany). The frozen slices were rewarmed and dried, fixed in fixing solution for 15 min, washed with distilled water, and dried. The slices were placed in the oil red dyeing solution protected from light and dipped for 8–10 min, and then the dye was washed off with distilled water. After that, 75% alcohol was used to differentiate and distilled water was used to wash away the alcohol. Counterstaining with hematoxylin for 3–8 min was performed, and the dye was washed away with distilled water. Hydrochloric acid and alcohol were used to quickly differentiate for 1 s; distilled water was used to wash away, the aqueous ammonia solution was used to return blue, and washing with distilled water was continued. Finally, glycerin gelatin was used to seal the tablets. The slice was scanned, and Image Pro Plus (version 6.0; Media Cybernetics, MD, USA) software was used to measure the oil red–stained area on the oil red–stained section of the mammary gland.

### Protein Identification and Label-Free Quantification

The mammary glands of five gilts from each treatment were randomly selected for proteomic analysis. Novogene Bioinformatics Technology in Beijing, China, was used to achieve it. Briefly, the sample was ground into powder in liquid nitrogen and mixed with a four times' volume of lysis solution (50 mM Tris-HCl, 8 M urea, 0.2% sodium dodecyl sulfate, pH 8). Then, samples were subjected to supersonic splitting thrice. Samples were centrifuged, and the precipitate was collected. The protein concentration was determined using a Bradford Protein Assay kit (ThermoFisher, Shanghai, China; Catalog No. 23236) according to the instructions.

Proteins were then digested with trypsin (37°C overnight) at an enzyme-to-protein ratio of 1:50. An equal volume of 1% formic acid to tryptic peptides was added, mixed, and centrifuged at room temperature at 12,000 *g* for 5 min. The supernatant was taken and slowly passed through a C18 desalting column. Then, 1 mL of cleaning solution (0.1% formic acid and 4% acetonitrile) was used to wash for three times. Then, 0.4 mL of eluent (0.1% formic acid, 45% acetonitrile) was added for two consecutive elutions. The eluted samples were combined and lyophilized.

Mobile phase solutions A (100% water, 0.1% formic acid) and B (80% acetonitrile, 0.1% formic acid) were prepared. The tryptic peptides were dissolved with 1 mL of solution A and separated by the EASY-nLC™ 1200 system (Thermo Scientific, Waltham, MA, USA). Then, they were analyzed on a Q Exactive™ HF-X mass spectrometer (MS) (Thermo Scientific). Full MS scans were performed at a scan range of 350 to 1,500 m/z and 3e6 C-trap. Data-dependent MS2 scans were monitored at a resolution of 15,000 and 1e5 C-trap using higher-energy collision dissociation with 27% of normalized collision energy and 60 s of dynamic exclusion time.

### Bioinformatics Analysis

Proteins were identified against the genome using the UniProt-GOA database (www.http://www.ebi.ac.uk/GOA/; Hinxton, England; P101SC18081032-01-sus-scrofa-uniprot-2018_8_2.fasta [48,936 sequences]) within Proteome Discoverer 2.2 (Thermo). Peptide spectrum match identification criteria included an identification probability of 95%. The protein containing at least one unique peptide is a trusted protein. Only the trusted spectrum peptides and proteins were retained, and false discovery rate (FDR) verification was done to remove peptides and proteins with FDR >5%. InterProScan (Hinxton, England) was used to annotate the corresponding Gene Ontology (GO) function of the differentially expressed proteins (DEPs) under the categories of biological process (BP), molecular function (MF), and cellular component (CC). We also identified and analyzed proteins within the KEGG (Kyoto Encyclopedia of Genes and Genomes) metabolic pathway (http://www.kegg.jp/).

### Quantitative Polymerase Chain Reaction Analysis

Specific parts of genes were selected to check the results obtained from the proteomics analysis. Total RNA was extracted with TRIzol reagent (Invitrogen, Carlsbad, CA, USA; Catalog No. 15596018) from frozen mammary gland tissue. The trace quantity DNA was removed by DNase-I (TaKaRa Biotechnology Co., Ltd., Dalian, China; catalog no. D2215), and the RNA was quantified by spectrophotometrically. cDNA was synthesized with random primers (Invitrogen, Carlsbad, CA, USA; catalog no. 48190-011). Real-time polymerase chain reaction was used to quantify the specific genes; the primers are listed in Supplementary Table 1. Amplification was carried out according to the product specifications (Takara, Tokyo, Japan). To get rid of the potential contamination, one reaction with the cDNA was replaced by water. Product sizes were verified by agarose gel electrophoresis, and all products were sequenced to confirm identity. Used as housekeeping gene, β-actin was amplified for each sample to verify the presence of cDNA and as an internal control to calculate the relative level of target gene expression using the 2^−Δ*ΔCt*^ method (28).

### Statistical Analysis

Before using parametric analyses, descriptive statistics were performed to check the normality and homogeneity of variances. All data were analyzed by two-way analysis of variance using Mixed Procedure of SAS 9.4 (SAS Institute, Cary, NC, USA) and GraphPad Prism 6.0 (GraphPad Inc., La Jolla, CA, USA; figures used). The statistical model was as follows: *Yijk* = μ + α*i* + β*j* + αβ*ij* + *eij*, where *Yijk* is the analyzed variable, μ is the overall mean, α*i* and β*j* are the effect of energy and fiber, αβ*ij* is the interaction effect, and *eij* is the residual error. When significant (*p* < 0.05) interactions were observed, the means were compared based on the least significant difference. The results are presented as mean ± SE. *p* < 0.05 was considered as a statistically significant difference. A trend was assumed at 0.05 ≤ *p* < 0.1.

To identify significant DEPs, the DEPs were first determined between the treatment and control from the fiber or energy, respectively. The proteins with a fold change (FC) of ≥1.2 (*p* < 0.05) was considered up-regulated, and those with FC < 0.82 (*p* < 0.05) were defined as down-regulated. Second, the relative FC (RFC) for the up-regulated and down-regulated proteins between energy or fiber was further compared, and the proteins with an RFC > 1.2 or < 0.82 were considered as significant up-regulated or down-regulated, respectively.

## Results

### Effect of Energy and Dietary Fiber on the Mammary Gland Development in Gilt

**Figure 1:**
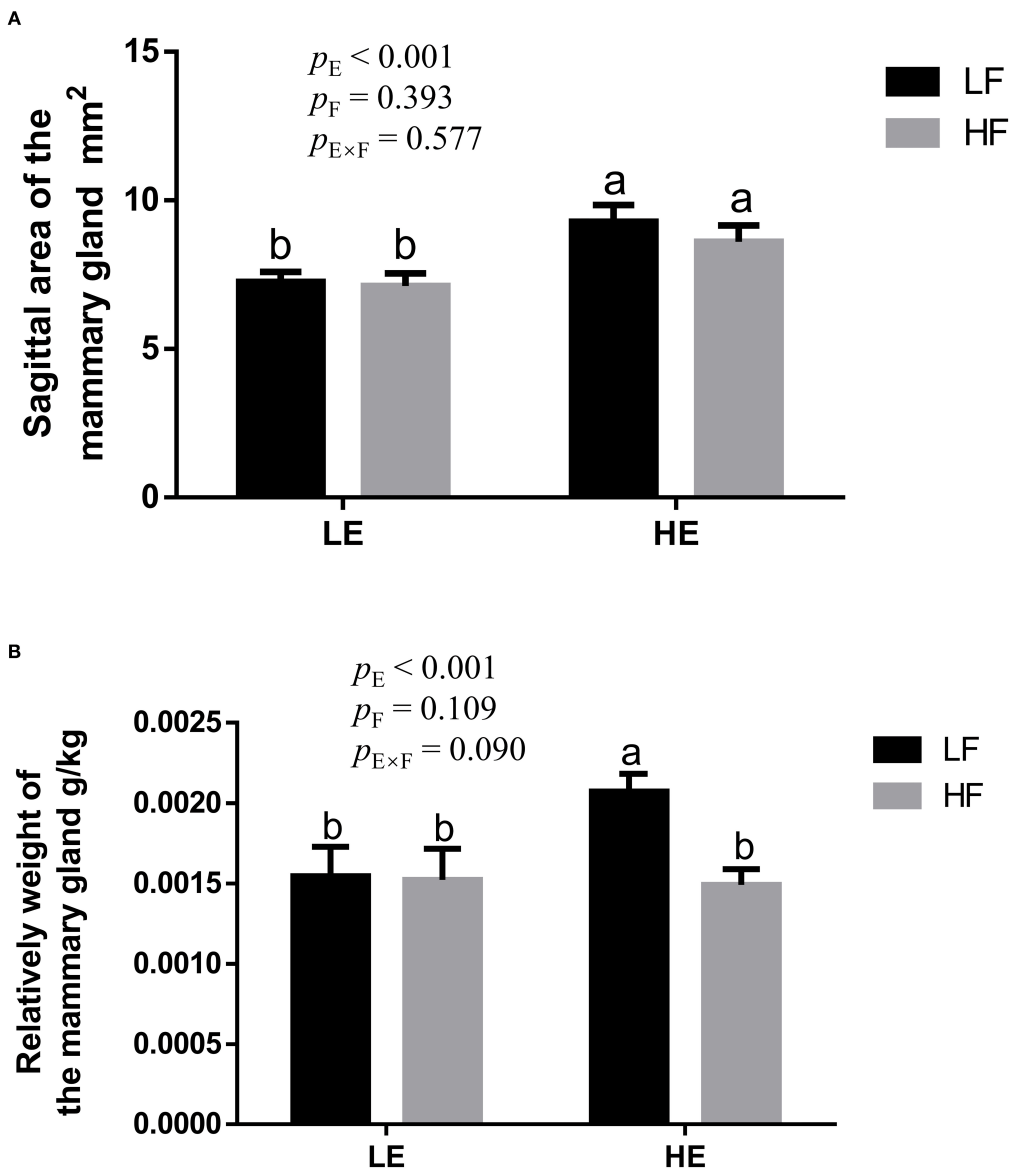
The effect of energy and fiber interaction on the sagittal area **(A)** and **(B)** relative weight of the mammary gland of the gilts (n = 8). LELF, low-energy low-fiber group; LEHF, low-energy high-fiber group; HELF, high energy low-fiber group; HEHF, high-energy high-fiber group; E × F, energy and fiber interaction. ^a, b^Means not sharing identical superscripts are significantly different (*p* < 0.05).

**Figure 2:**
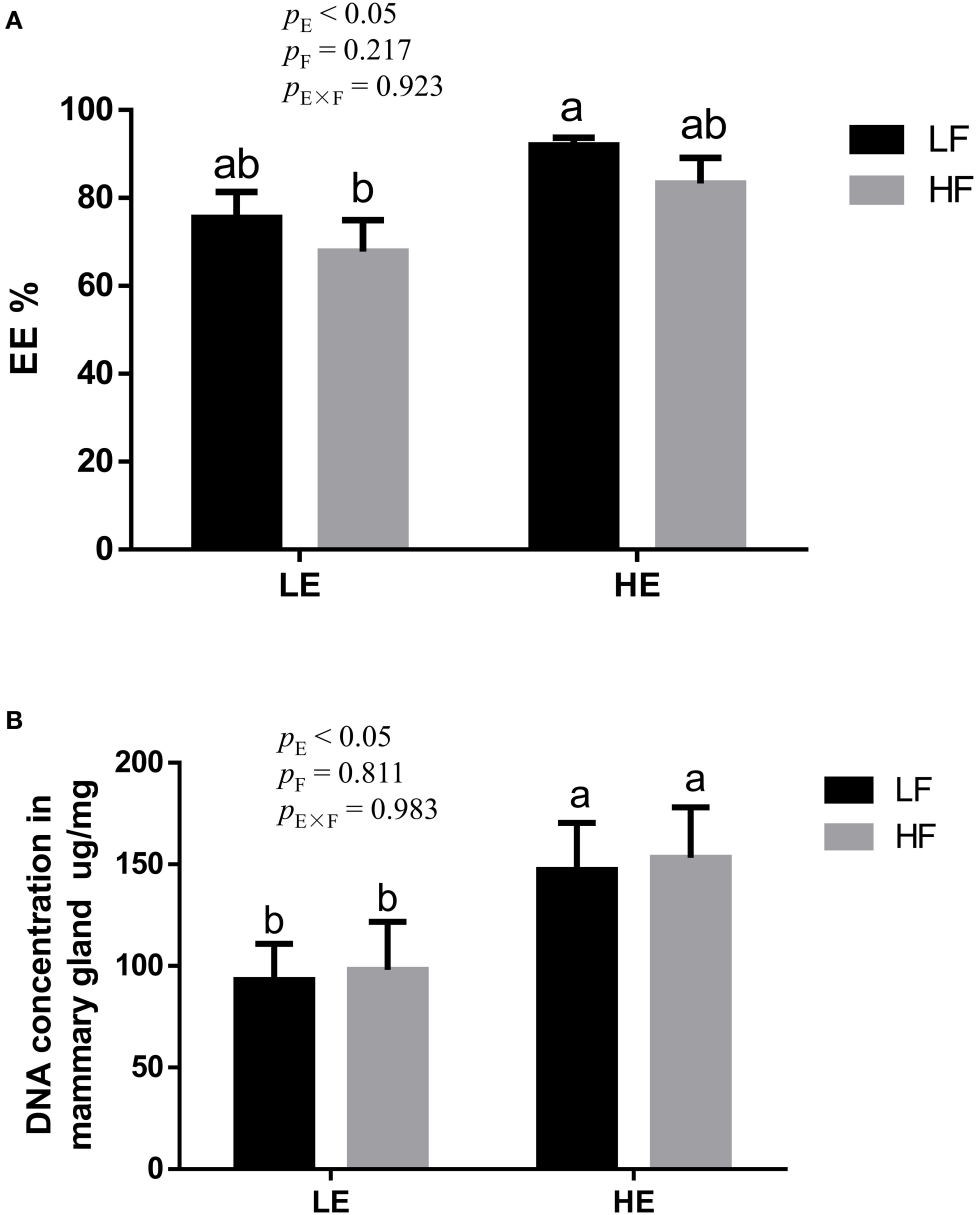
Effects of energy and fiber interaction on crude fat **(A)** and DNA **(B)** concentration in mammary gland of gilts (n = 8). LELF, low-energy low-fiber group; LEHF, low-energy high-fiber group; HELF, high-energy low-fiber group; HEHF, high-energy high-fiber group; E × F, energy and fiber interaction. ^a, b^Means not sharing identical superscripts are significantly different (*p* < 0.05).

**Figure 3:**
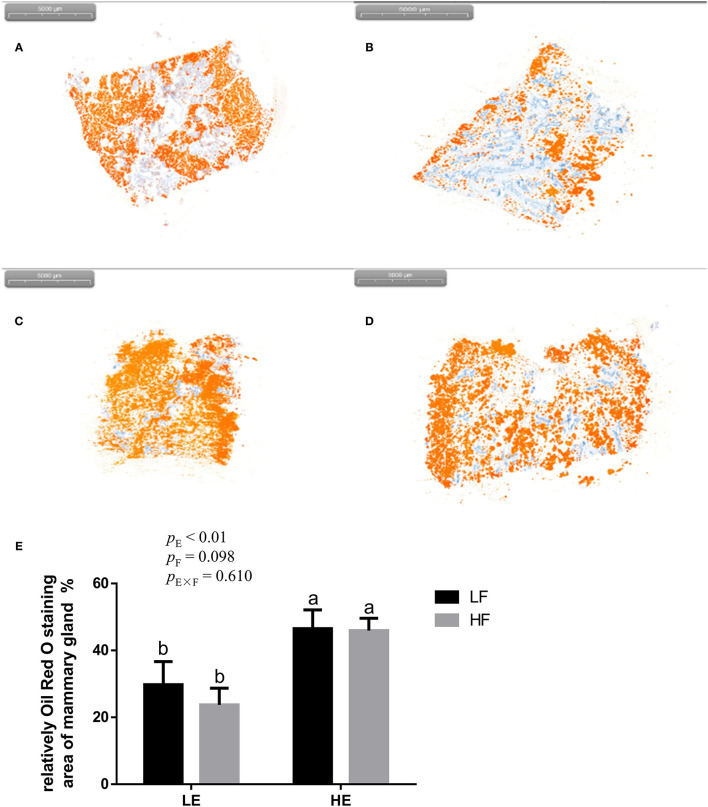
Representative images **(A–D)** of red-staining sections of mammary gland in gilts and the effect of energy and fiber on the relatively oil red O–staining area of mammary gland in gilts (n = 8, **E**). **(A)** Low-energy low-fiber group; **(B)** low-energy high fiber; **(C)** high-energy low-fiber group; **(D)** high-energy high-fiber group. LELF, low-energy low-fiber; LEHF, low-energy high-fiber; HELF, high-energy low-fiber; HEHF, high-energy high-fiber; E × F, energy and fiber interaction. ^a, b^Means not sharing identical superscripts are significantly different (*p* < 0.05).

High energy intake significantly increased the sagittal area and relative weight of mammary glands in the gilts (*p* < 0.05; Figure 1). However, under high-energy conditions, high fiber intake decreased the relative weight of mammary glands (*p* < 0.05; Figure 1B). As shown in Figure 2, high energy intake increased the EE and DNA concentration in mammary gland tissue of gilts (*p* < 0.05). The relative area of oil red O staining of breast tissue of gilts was increased by the high energy intake (*p* < 0.01); the high fiber intake tended to decrease the area of oil red O staining of breast tissue (*p* = 0.098; Figure 3).

### Effect of Energy and Dietary Fiber on Plasma Metabolites Parameters in Gilt

High energy intake increased the concentration of TGs, free fatty acids, and TC in the blood of gilts (*p* < 0.05; Table 1). High dietary fiber intake showed a tendency to reduce the concentration of free fatty acids (*p* = 0.061) and TC (*p* = 0.088) in the blood circulation. The high energy and high dietary fiber intake showed an interaction effect on plasma free fatty acid content (*p* = 0.039).

**Table 1:** Effects of energy and fiber levels on plasma metabolites in gilts.

**(mmol/L)**	**LE**		**HE**		***p*** **-Value**		
	**LF**	**HF**	**LF**	**HF**	**Energy**	**Fiber**	**E × F**
TG	0.291 ± 0.027^b^	0.283 ± 0.026^b^	0.416 ± 0.042^a^	0.330 ± 0.027^a^^b^	0.010	0.142	0.219
TC	1.896 ± 0.144^b^	1.825 ± 0.057^b^	2.244 ± 0.072^a^	1.979 ± 0.082^a^^b^	0.013	0.088	0.315
NEFA	0.082 ± 0.006^c^	0.085 ± 0.008^c^	0.205 ± 0.021^a^	0.150 ± 0.014^b^	<0.001	0.061	0.039
HDL-C	0.845 ± 0.077	0.834 ± 0.032	0.845 ± 0.061	0.916 ± 0.065	0.501	0.625	0.507
LDL-C	0.879 ± 0.075	0.794 ± 0.022	0.916 ± 0.062	0.800 ± 0.062	0.714	0.100	0.800

*Values are mean ± SE. n = 8. TG, triglycerides; TC, total cholesterol; NEFA, nonesterified fatty acid; HDL-C, high-density lipoprotein cholesterol; LDL-C, low-density lipoprotein cholesterol; LE, low energy; HE, high energy; LF, low fiber; HF, high fiber; E × F, energy and fiber interaction*.

a *,*

b *Means not sharing identical superscripts in the same row are significantly different (p < 0.05)*.

### Effect of Energy and Dietary Fiber on Serum Hormone Concentration in Gilt

High energy intake increased the concentration of serum estrogen in gilts (*p* < 0.05; Table 2). Different energy and fiber intake levels did not affect the concentration of prolactin, testosterone, and progesterone in the serum (*p* > 0.05).

**Table 2:** Effects of energy and fiber levels on serum hormone concentrations in gilts.

**Items**	**LE**		**HE**		***p*** **-Value**		
	**LF**	**HF**	**LF**	**HF**	**Energy**	**Fiber**	**E × F**
Estradiol, pg/mL	71.14, 6.36^a^^b^	55.14, 5.59^b^	94.58, 14.39^a^	87.21, 12.79^a^^b^	0.013	0.276	0.685
Prolactin, mIU/L	228.00, 12.90	249.00, 11.50	244.20, 14.90	244.50, 3.00	0.599	0.342	0.359
Testosterone, pg/mL	73.50, 3.30	78.20, 3.30	78.00, 2.60	78.10, 2.50	0.467	0.427	0.436
Progesterone, ng/mL	2.63, 0.19	3.26, 0.28	2.88, 0.34	3.03, 0.21	0.969	0.156	0.385

*Values are mean ± SE. n = 8. LE, low energy; HE, high energy; LF, low fiber; HF, high fiber; E × F, energy and fiber interaction*.

a *,*

b *Means not sharing identical superscripts in the same row are significantly different (p < 0.05)*.

### DEP Analysis in Proteomics

Proteins with an FC > 1.2 (*p* < 0.05) between the treatments were considered up-regulated, whereas proteins with an FC < 0.82 (*p* < 0.05) were considered down-regulated. Based on these criteria, there were 53 proteins up-regulated, whereas 58 proteins down-regulated on the main effects of energy (Supplementary Table 2). There were 25 proteins up-regulated and 6 proteins down-regulated on the main effects of fiber (Supplementary Table 3). Under the interaction of energy and fiber, there were 10 proteins up-regulated and 10 proteins down-regulated (Supplementary Table 4). Among them, the proteins that affect the development of mammary glands of gilts included lipid metabolism, reduction metabolism, mitochondrial respiratory chain, molecular chaperone that maintains the normal function of the protein, and energy metabolism (Table 3).

**Table 3:** Differentially expressed proteins (DEPs) associated with development of mammary glands in the energy and fiber treatments in gilt.

**Protein accession**	**Protein description**	**FC/energy**	**FC/fiber**	**Gene name**
F1RZK8	Histone deacetylase	5.022224	—	*HDAC2*
A0A287BM29	Apolipoprotein A-IV	3.603054	—	*APOA4*
A0A0D5BWD2	Mitochondrial complement component 1 Q subcomponent-binding protein	1.785695	—	*C1QBP*
F1SLR1	NADH dehydrogenase	0.463774	—	*NDUFA8*
P12309	Glutaredoxin-1	0.458297	—	*GLRX*
A0A287A808	Cytochrome c oxidase subunit	0.437125	—	*COX6B*
P27917	Apolipoprotein C-III	—	1.609517	*APOC3*
F1S0J2	Apolipoprotein R precursor	—	1.51511	*C4BPA*
A0A287AH85	Adenylate kinase 2, mitochondrial	—	0.81694	*AK2*
I3LNG8	Stress-induced phosphoprotein 1	—	0.726254	*STIP1*
A0A287BEZ5	Acetyl-coenzyme A synthetase, cytoplasmic	—	0.533272	*ACSS2*
F1SIS9	NADH dehydrogenase [ubiquinone] 1α subcomplex subunit 10, mitochondrial	—	0.380405	*NDUFA10*

*The change of protein expression level was expressed by the ratio of energy or fiber/control group. The FC (fold change) ratio >1 (p < 0.05) indicates up-regulation, and the FC ratio < 1 (p < 0.05) indicates down-regulation*.

### Functional Enrichment Analysis of DEPs

**Figure 4:**
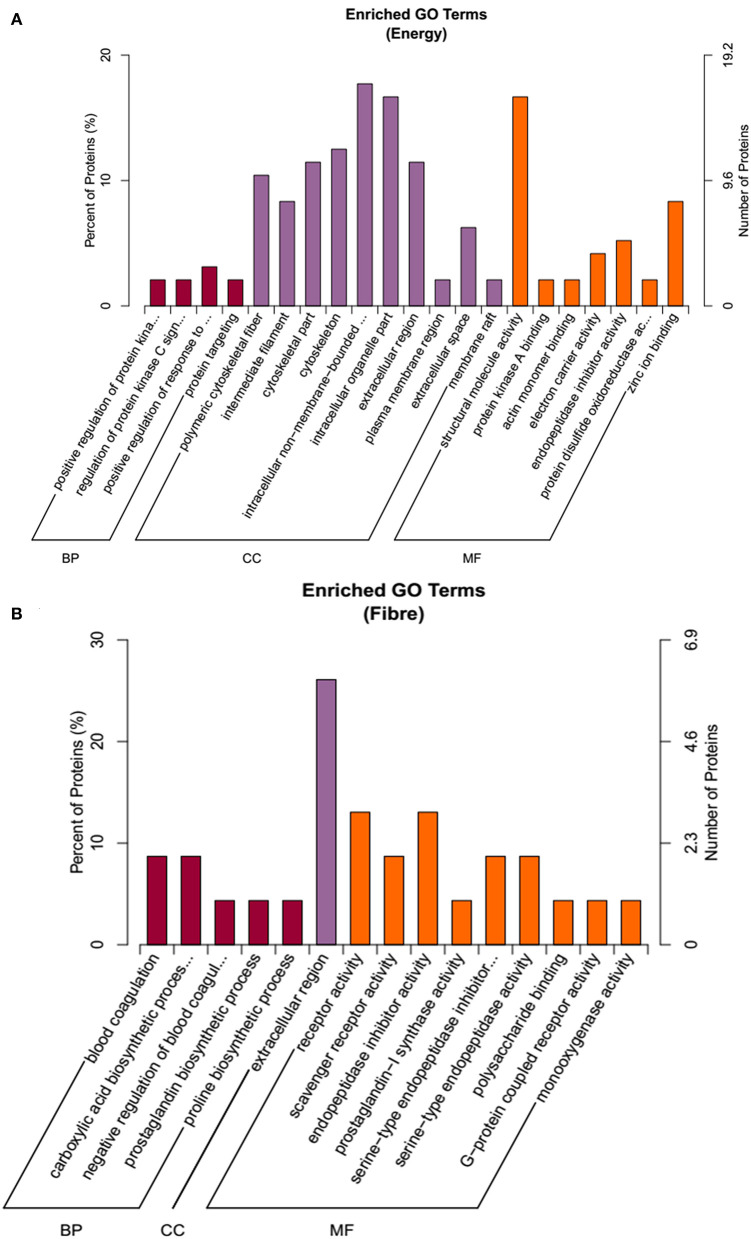
The functional enrichment of Gene Ontology (GO) annotation of the DEPs. **(A)** Energy differential protein GO annotation diagram. **(B)** Fiber differential protein GO annotation diagram.

To annotate the function of the treatments response proteins, protein IDs were searched against the Uniprot database (http://www.uniprot.org/). From the GO term annotation, the DEPs on the main effects of energy were enriched in BP, CC, and MF (Figure 4A). BP included protein metabolic processes, and CC mainly included cytoskeleton, whereas MF included structural molecular activity, zinc iron binding, endopeptidase inhibitor activity, and so on. For the fiber response, proteins were involved in CC, BP, and MF, including carboxylic acid biosynthetic process, prostaglandin biosynthetic process, extracellular region, G-protein–coupled receptor activity, and so on (Figure 4B).

### Effect of Energy and Dietary Fiber on Relative mRNA Expression of Mammary Gland in Gilt

High energy intake significantly increased the relative mRNA expression of 5-hydroxytryptamine receptor 7 (5-HT7) in mammary gland of gilts (*p* < 0.05; Table 4). High energy intake also increased the mRNA expressions of apoptosis relative gene B-cell lymphoma protein 2–associated X protein (*Bax*) and *caspase-3* in mammary gland of gilts (*p* < 0.05).

**Table 4:** Effects of energy and fiber levels on relative mRNA expression in mammary gland.

**Items**	**LE**		**HE**		***p***-**Value**		
	**LF**	**HF**	**LF**	**HF**	**Energy**	**Fiber**	**E × F**
*5-HT_1*D*_*	1.00 ± 0.12	1.18 ± 0.20	1.41 ± 0.16	1.31 ± 0.12	0.087	0.777	0.367
*5-HT_2*A*_*	1.00 ± 0.61	1.11 ± 0.99	1.93 ± 1.92	1.34 ± 0.97	0.076	0.458	0.277
*5-HT_2*B*_*	1.00 ± 0.13	1.43 ± 0.30	1.59 ± 0.59	1.50 ± 0.51	0.108	0.389	0.214
*5-HT_7_*	1.00 ± 0.09^b^	1.08 ± 0.12^b^	1.42 ± 0.17^a^	1.40 ± 0.19^a^	0.044	0.838	0.765
*BAX*	1.00 ± 0.06^b^	1.03 ± 0.09^b^	1.47 ± 0.26^a^	1.35 ± 0.19^a^	0.029	0.801	0.674
*BCL-2*	1.00 ± 0.12	0.76 ± 0.05	0.91 ± 0.15	0.91 ± 0.13	0.818	0.315	0.322
*CAS-3*	1.00 ± 0.19^b^	1.07 ± 0.21^b^	1.47 ± 0.20^a^	1.51 ± 0.20^a^	0.030	0.780	0.941

*Values are mean ± SE. n = 8. LE, low energy; HE, high energy; LF, low fiber; HF, high fiber; E × F, energy and fiber interaction; 5-HTR_1D_, 5-hydroxytryptamine receptor 1D; 5-HTR_2A_, 5-hydroxytryptamine receptor 2A; 5-HT_2B_, 5-hydroxytryptamine receptor 2B; 5-HT_7_, 5-hydroxytryptamine receptor 7; BAX, B-cell lymphoma protein 2–associated X protein; BCL-2, B-cell leukemia-2; CAS-3, caspase-3*.

a *,*

b *Means not sharing identical superscripts in the same row are significantly different (p < 0.05)*.

## Discussion

Our previous study found that high-energy diets increased the thickness of back fat and advanced the estrus age of gilts; at the same time, high-fiber diet intake counteracted the excessive antral follicular atresia caused by high energy intake (24). It is reported that with the increase in diet energy, the concentration of free fatty acids increased in the blood of gilts (29). As the concentration of free fatty acids in the blood increased, the cholesterol content that transports fatty acids also tended to increase (30). In the present study, the results showed that a high-energy diet significantly increased the concentration of TGs, free fatty acids, and TC in the blood; increased sagittal area and the relative weight of mammary glands; and increased the EE of mammary gland tissue of gilts. Moreover, after oil red O staining was performed on the sections of the mammary gland, it was found that high-energy diet could significantly increase the oil red area (the area of oil red O staining represents the fat content). Furthermore, it was found in this study that high energy intake resulted in an up-regulation of apolipoprotein A-IV in proteomic analysis. Apolipoprotein A-IV plays an important role in lipoprotein metabolism and maintenance of lipid levels (31). As previous results have shown that high energy intake significantly increases blood levels of TGs, free fatty acids, and TC, it is speculated that the up-regulation of apolipoprotein A-IV due to high energy intake may be an adjustment response to the high energy intake of gilts. These results suggest that the weight and size of the mammary gland can be affected by increasing the fat deposition in the mammary gland of the gilts under high-energy conditions. In this study, high fiber intake reduced the relative weight of the mammary glands under high-energy conditions. One study has shown that the addition of dietary fiber to the diet of growing and fattening pigs reduced fat deposition and increased the carcass lean meat rate (29). These indicate that the high-fiber diet reduced the fat deposition in the mammary gland of gilts and reduced the burden of high-energy fat deposition in the mammary gland.

Studies have confirmed that estrogen plays an important role in the ductal extension during puberty and acinar development during pregnancy (32). During the puberty of gilt, estrogen increased the blood flow speed of blood vessels by increasing the permeability of cell membranes and the accumulation of extracellular fluid, thereby promoting the synthesis of DNA and protein and thus stimulating the development of mammary duct system (33). It had found that estradiol secretion was influenced by cholesterol level and positively correlated with body fat tissue content (34). The results of this study suggest that increasing diet energy promotes the secretion of estrogen, which may in turn promote ductal extension of mammary during puberty. The present results show that a high-energy diet significantly increased DNA concentration in mammary gland tissue of gilts. The amount of mammary tissue, DNA, and RNA was positively correlated to the average daily gain of the piglets in the lactation period (35). The increase in DNA concentration indicated that high energy intake increased the activity of mammary cells. However, studies have shown that high energy intake during pregnancy has side effects on the development of the mammary glands and subsequent lactation (12, 13). A study has found that the increase in free fatty acids inhibited the growth of mammary epithelial cells in rats (36). Exogenous free fatty acid challenge induced increase in concentrations of mitochondrial reactive oxygen species (ROS) and cell apoptosis in bovine mammary epithelial cells (37). In addition, it also found a significant increase in the cell apoptosis treated with free fatty acids in human vascular endothelial cells (38). These findings suggest that free fatty acids can promote apoptosis. In this study, high-energy diet–induced increase in free fatty acid concentration in the blood of gilts may promote cell apoptosis of the mammary gland. However, high-fiber supplementation reduced the increase in free fatty acid concentration in the blood of gilts caused by high-energy diet in this study, which may reduce the promotion of fatty acid on cell apoptosis.

The DEPs on the main effects of energy were enriched in BP, CC, and MF, including protein metabolic processes, cytoskeleton, structural molecular activity, zinc iron binding, endopeptidase inhibitor activity, and so on. For the fiber response, proteins were involved in CC, BP, and MF, including carboxylic acid biosynthetic process, prostaglandin biosynthetic process, extracellular region, G-protein–coupled receptor activity, and so on. Glutaredoxin (Grx) has a variety of biological activities and plays an important role in regulating redox reaction and cell growth and inhibiting apoptosis (39). Grx has been found to inhibit oxidative stress damage in cells by mediating the neurotransmitter dopamine through the nuclear factor κB pathway. Moreover, Grx system can prevent and treat ROS-induced oxidative stress injury. The results of this study showed that under high energy intake, the expression level of Grx in the mammary gland of gilts decreased, which indicated that the antioxidant stress and antiapoptosis ability of mammary cells decreased. This result was confirmed by the gene expression result, which showed that the expression of proapoptotic genes (*Bax* and *caspase-3*) increased with high energy intake. It has been reported that 5-HT7 is involved in the regulation of breast epithelial tight junctions (40). As 5-HT secreted by mammary epithelial cells can regulate breast development and milk synthesis, 5-HT7–mediated 5-HT plays an important role in breast epithelial cell shedding and cell death (41). The results of this study showed that high energy intake increased mRNA expression of *5-HT7* in mammary gland of gilts. Therefore, the high energy intake weakened the antiapoptotic ability of the gilts' mammary gland. Cytochrome C oxidase (COX) is a terminal complex of electron transport in the mitochondrial respiratory chain and a key regulatory site of mitochondrial oxidation capacity (42). Studies have shown that COX is involved in the regulation of apoptosis by regulating mitochondrial electron transfer, mitochondrial membrane potential, and energy synthesis (43). In this study, the proteomic results showed that the expression of the COX subunit in the gilt breast cells was down-regulated under high energy intake, indicating an increase in apoptosis of breast cells. This result may be due to the increase in oxidative stress caused by high energy, resulting in a decrease in the number of COX subunits.

Stress-induced phosphorylated protein 1 (STIP1) has a regulatory effect on a variety of molecular proteins and is widely involved in cell gene transcription, signal transduction, and proliferation and division (44, 45). In addition, STIP1 can regulate cell growth, inhibit cell apoptosis, regulate some active proteins in the body by regulating many downstream molecular proteins, and participate in multiple metabolisms by acting as a molecular chaperone or complex molecular chaperone (46, 47). In this experiment, it was found that intake of high fiber led to the down-regulated expression of STIP1 in gilts. Therefore, we speculated that high fiber may reduce cell proliferation by reducing STIP1 expression. Adenylate kinase (AK) is mainly focused on maintaining the balance of energy supply in living organisms (48). However, a recent study found that AK2 was associated with apoptosis, during which cytochrome C and AK2 were transferred to the cytoplasm together (49). Further studies showed that the apoptotic factor Bax could lead to the release of AK2, and the release amount was positively correlated with the concentration of Bax (50). In this study, high energy intake significantly increased mRNA expression of apoptosis-related genes (*Bax* and *caspase-3*) in mammary gland of gilts, and it was also found that the expression of AK2 was down-regulated because of high energy and high fiber intake, suggesting that AK2 may inhibit cell apoptosis after the addition of high fiber to high energy. It is speculated that the addition of high fiber can alleviate the apoptosis of breast cells brought by high energy to a certain extent.

In conclusion, the current results suggest that high energy intake of gilts increased breast fat deposition, and mammary proteomics analysis showed that high-energy diets increased mammary gland cell apoptosis. Increased fiber intake (inulin and cellulose, ratio 1:4) in gilts counteracted the breast fat deposition and the apoptosis of breast cells induced by high energy intake. The beneficial improvement of mammary gland for gilts may be due to the regulation of key points in lipid metabolism, the reduction metabolism, and mitochondrial respiratory chain system.

## Data Availability Statement

The original contributions presented in the study are included in the article/Supplementary Material, further inquiries can be directed to the corresponding authors.

## Ethics Statement

The animal study was reviewed and approved by Animal Care and Use Committee of Sichuan Agricultural University (Permit Number SICAU2015034).

## Author Contributions

LT and MC carried out the animal experiments and performed the laboratory work. YY, JL, YL, ZF, YZ, HX, and LC performed the statistical analysis. SX, BF, YZ, JW, and DW conceived and designed the experiment. SX and SC wrote the paper. All authors critically reviewed the manuscript and gave final approval for the version to be published.

## Funding

The present study was funded by Sichuan Province 145 Breeding Tackle Project (2021YFYZ0008) and Sichuan Agricultural University Double Support Project.

## Conflict of Interest

The authors declare that the research was conducted in the absence of any commercial or financial relationships that could be construed as a potential conflict of interest.

## Publisher's Note

All claims expressed in this article are solely those of the authors and do not necessarily represent those of their affiliated organizations, or those of the publisher, the editors and the reviewers. Any product that may be evaluated in this article, or claim that may be made by its manufacturer, is not guaranteed or endorsed by the publisher.
