# Supplementary material for: Effects of Energy and Dietary Fiber on the Breast Development in Gilt
Source: Front Vet Sci. 2022 Mar 10;9:830392. doi: 10.3389/fvets.2022.830392 (PMC8960423; doi:10.3389/fvets.2022.830392)
Supplement: Supplementary file 1 [file Table_1.DOCX]

**Supplementary data**

**Supplemental tables**

**Supplemental Table 1.** Oligonucleotide primers used for a relative-quantitative real-time PCR analysis

| Gene name | Forward Sequence (5′$\to$3′) | Reverse Sequence (5′$\to$3′) | Accession No. |
| --- | --- | --- | --- |
| *BAX* | CGCATTGGAGATGAACTGGA | CCAGTTGAAGTTGCCGTCAG | XM_003127290.5 |
| *BCL-2* | GCCTTTGTGGAGCTGTATGG | CCCGTGGACTTCACTTATGG | XM_021099593.1 |
| *CAS-3* | GCCGAGGCACAGAATTGGACTG | GCCAGGAATAGTAACCAGGTGCTG | NM_214131.1 |
| *5-HTR_1D_* | ATCCAGGGACCCTCCAAGTC | AGTGTGGAGCTTCCTGGTCA | NM_214158.1 |
| *5-HTR_2A_* | TCTCTCACCACCCTGCTTCT | TCTCTAGGGACACTGCCATGA | NM_214217.1 |
| *5-HTR_2B_* | CGCCTAACATGGTTGACTGTG | TGGGCAGAGTTTTGTCCTTGT | NM_001164019.1 |
| *5-HTR_7_* | CCGTCAGGCAGAATGGCAAG | TCGTCATTTACGTTCTGCGCC | NM_214085.1 |
| *β-actin* | CCAGCACGATGAAGATCAAGA | AATGCAACTAACAGTCCGCCTA | XM_003124280.5 |

*BAX*, B-cell lymphoma protein 2 associated X protein; *BCL-2*, B-cell leukaemia-2; *CAS-3*, *Caspase-3*; *5-HTR_1D_*, 5-hydroxytryptamine receptor 1D; *5-HTR_2A_*, 5-hydroxytryptamine receptor 2A; *5-HT_2B_*, 5-hydroxytryptamine receptor 2B; *5-HT_7_*, 5-hydroxytryptamine receptor 7.

**Supplemental Table 2.** High-energy diet intake up-regulated or down-regulated the mammary gland protein expression in gilts

| **Protein Accession** | **Protein Description** | **Gene Name** | **FC** |
| --- | --- | --- | --- |
| F1RZK8 | Histone deacetylase | *HDAC2* | 5.022224 |
| A0A287BM29 | Apolipoprotein A-IV | *APOA4* | 3.603054 |
| I3LUI5 | Family with sequence similarity 151 member B | *FAM151B* | 3.466937 |
| A0A075B7I5 | Uncharacterized protein |  | 3.427086 |
| F1RK90 | Periplakin | *PPL* | 2.351382 |
| F1SUW3 | EF-hand domain family member D2 | *EFHD2* | 2.285676 |
| F1S0K2 | Keratin 15 | *KRT15* | 2.03271 |
| B2ZPK1 | Cytochrome b reductase 1 | *CYBRD1* | 1.94137 |
| K7GMF6 | Adhesion G protein-coupled receptor E5 | *ADGRE5* | 1.940473 |
| F6PU32 | Calpastatin | *CAST* | 1.923755 |
| A0A287AC69 | Proliferation and apoptosis adaptor protein 15 | *PEA15* | 1.912255 |
| F1RWP3 | Cytoglobin | *CYGB* | 1.891136 |
| F1S6B5 | Fibromodulin | *FMOD* | 1.87629 |
| F1RQZ0 | Granulins precursor | *GRN* | 1.807257 |
| A0A0D5BWD2 | Mitochondrial complement component 1 Q subcomponent-binding protein | *C1QBP* | 1.785695 |
| I3LEI8 | 1,2-dihydroxy-3-keto-5-methylthiopentene dioxygenase | *ADI1* | 1.752536 |
| F1RM40 | Symplekin | *SYMPK* | 1.752164 |
| P02540 | Desmin | *DES* | 1.708037 |
| A0A287BRY0 | Tropomyosin alpha-1 chain | *TPM1* | 1.701739 |
| I3LDR9 | Caveolae associated protein 2 | *CAVIN2* | 1.693877 |
| A5A779 | Geranylgeranyl transferase type-2 subunit alpha | *RABGGTA* | 1.6861 |
| A0A286ZVB7 | Dermatopontin | *DPT* | 1.646752 |
| Q9XSD9 | Decorin | *DCN* | 1.588323 |
| A0A287AFG3 | Lymphocyte-specific protein 1 | *LSP1* | 1.573594 |
| A0A287A3A3 | A-kinase anchoring protein 12 | *AKAP12* | 1.567035 |
| I3LFR2 | Cullin 5 | *CUL5* | 1.523849 |
| A0A287A6T5 | Tropomyosin alpha-1 chain | *TPM1* | 1.510519 |
| P21753 | Thymosin beta-10 | *TMSB10* | 1.507662 |
| F1SD87 | Fibulin 5 | *FBLN5* | 1.487471 |
| A0A287B600 | Synemin | *SYNM* | 1.478385 |
| F1S1U5 | Golgi reassembly stacking protein 2 | *GORASP2* | 1.477179 |
| A0A287AT05 | Serum albumin | *ALB* | 1.454385 |
| A0A287AUQ9 | A-kinase anchoring protein 12 | *AKAP12* | 1.416985 |
| F1SL54 | Coatomer subunit beta | *COPB2* | 1.400042 |
| B7TJ02 | Thymosin beta 4 X-linked | *TMSB4X* | 1.396065 |
| A0A287BM82 | Keratin 14 | *KRT14* | 1.391129 |
| F1RYZ0 | 60S acidic ribosomal protein P2 | *RPLP2* | 1.387075 |
| F1SG00 | Tropomyosin beta chain | *TPM2* | 1.383205 |
| F1S0J8 | Keratin 14 | *KRT14* | 1.357263 |
| K9J4R3 | Alpha-mannosidase | *MAN2B1* | 1.341733 |
| A0A221ZTU1 | MHC class II antigen | *DQB1* | 1.327651 |
| A0A286ZN89 | Uncharacterized protein |  | 1.293096 |
| A0A287B0I3 | Rho GDP-dissociation inhibitor 2 | *ARHGDIB* | 1.276642 |
| F1SV59 | ADP-ribosylhydrolase like 2 | *ADPRHL2* | 1.269552 |
| F1SQ01 | Peroxiredoxin 4 | *PRDX4* | 1.266331 |
| Q66X52 | Ragulator complex protein LAMTOR5 | *LAMTOR5* | 1.264313 |
| F1SGG1 | Keratin 18 | *KRT18* | 1.260607 |
| F1SGT4 | CD44 molecule (Indian blood group) | *CD44* | 1.259283 |
| F1S9A4 | Uncharacterized protein | *NUCB2* | 1.249709 |
| A0A287AEL2 | Keratin 14 | *KRT14* | 1.242625 |
| A0A287BKE2 | N-myc downstream regulated 1 | *NDRG1* | 1.241460 |
| A0A286ZWS0 | SH3 domain-binding glutamic acid-rich-like protein | *SH3BGRL3* | 1.209964 |
| A0A287BGV1 | Latent transforming growth factor beta binding protein 4 | *LTBP4* | 1.202825 |
| A5A759 | Keratin 2A | *KRT2A* | 0.258854 |
| A0A287BEZ5 | Acetyl-coenzyme A synthetase, cytoplasmic | *ACSS2* | 0.314623 |
| A0A287B781 | Uncharacterized protein | *LOC110258364* | 0.340046 |
| F6Q469 | Alpha-1-antichymotrypsin 2 precursor | *SERPINA3-2* | 0.355009 |
| A0A287BL80 | Mannose-6-phosphate isomerase | *MPI* | 0.406479 |
| A0A287ATB9 | Uncharacterized protein |  | 0.433801 |
| A0A287A808 | Cytochrome c oxidase subunit | *COX6B* | 0.437125 |
| A0A287B0Z5 | IgG receptor FcRn large subunit p51 precursor | *FCGRT* | 0.443524 |
| P12309 | Glutaredoxin-1 | *GLRX* | 0.458297 |
| A0A286ZQY9 | Ribulose-phosphate 3-epimerase | *RPE* | 0.45879 |
| F2Z5C8 | Signal peptidase complex subunit 3 | *SPCS3* | 0.459852 |
| F1SLR1 | NADH dehydrogenase | *NDUFA8* | 0.463774 |
| D0G781 | Acyl carrier protein | *NDUFAB1* | 0.47641 |
| F1RM15 | Leucyl-tRNA synthetase | *LARS* | 0.491279 |
| K7GSI9 | Four and a half LIM domains protein 1 | *FHL1* | 0.50821 |
| I3LKM9 | Legumain | *LGMN* | 0.52193 |
| F1RPU8 | Mitochondrial pyruvate carrier | *MPC2* | 0.530192 |
| I3LL97 | Cysteine and glycine rich protein 1 | *CSRP1* | 0.542346 |
| F1RJI1 | Malectin | *MLEC* | 0.545647 |
| A0A287B3F4 | Amylo-alpha-1, 6-glucosidase, 4-alpha-glucanotransferase | *AGL* | 0.548792 |
| A5A8V8 | U6 snRNA-associated Sm-like protein LSm2 | *LSM2* | 0.553728 |
| A0A287B8A4 | Kelch repeat and BTB domain containing 11 | *KBTBD11* | 0.553918 |
| A0A165DE67 | CRIP2 | *CRIP2* | 0.553918 |
| F1RX84 | Pyridoxal phosphate homeostasis protein | *PLPBP* | 0.557128 |
| A0A287BHL0 | Poly(rC)-binding protein 2 | *PCBP2* | 0.564122 |
| P63221 | 40S ribosomal protein S21 | *RPS21* | 0.567072 |
| A0A287AME4 | Ribosomal protein L37a | *RPL37A* | 0.572765 |
| A0A287BPW3 | Uncharacterized protein | *LOC110258651* | 0.601769 |
| B3STX9 | Prothrombin |  | 0.608383 |
| L7PBE6 | T-complex protein 1 subunit epsilon | *CCT5* | 0.609508 |
| A0A287AH53 | Four and a half LIM domains 2 | *FHL2* | 0.610742 |
| A0A287BD93 | 2,3-cyclic-nucleotide 3-phosphodiesterase | *CNP* | 0.612461 |
| K7GP63 | Uncharacterized protein | *RPL39* | 0.619816 |
| F1RP44 | Importin-5 | *IPO5* | 0.638609 |
| F1S3H9 | Uncharacterized protein | *LOC100517145* | 0.639024 |
| A0A286ZPD7 | Transmembrane emp24 domain-containing protein 7 precursor | *TMED7* | 0.654107 |
| Q8WNV7 | Dehydrogenase/reductase SDR family member 4 | *DHRS4* | 0.657958 |
| A0A287AG13 | Apolipoprotein B-100 | *APOB* | 0.667126 |
| A1XQV6 | COX7A2 | *COX7A2* | 0.670794 |
| A0A287AQ67 | 40S ribosomal protein S29 | *RPS29* | 0.678043 |
| A0A287BRH5 | Tubulin gamma chain | *TUBG2* | 0.678106 |
| A0A287AIE6 | ATPase ASNA1 | *ASNA1* | 0.679241 |
| B0FWK6 | Aminoacyl tRNA synthase complex-interacting multifunctional protein 1 | *SCYE1* | 0.685614 |
| A0A286ZW72 | 60S ribosomal protein L14 | *RPL14* | 0.691727 |
| I3LJA6 | Uncharacterized protein |  | 0.694509 |
| F1S1X3 | Asparaginyl-tRNA synthetase | *NARS* | 0.695041 |
| A0A287BPL8 | Cadherin-1 precursor | *CDH1* | 0.725661 |
| I3LUM8 | Uncharacterized protein | *FARSB* | 0.739195 |
| F1SHC1 | Tubulin alpha chain | *TUBA1C* | 0.750642 |
| F1SBS4 | Complement C3 | *C3* | 0.75286 |
| A0A287AY28 | Cytoplasmic dynein 1 intermediate chain 2 | *DYNC1I2* | 0.765358 |
| I3LRJ4 | Vitamin K-dependent protein C | *PROC* | 0.766491 |
| A0A287AQ20 | Uncharacterized protein | *CFI* | 0.77012 |
| A0A287BDU7 | Uncharacterized protein | *PZP* | 0.781466 |
| A0A286ZSJ7 | Complement C1q subcomponent subunit C precursor | *C1QC* | 0.785267 |
| A0A287A4W6 | RAB21, member RAS oncogene family | *RAB21* | 0.789437 |
| A0A287A1B4 | Uncharacterized protein | *PZP* | 0.789579 |
| F1S8H8 | Purine nucleoside phosphorylase | *PNP* | 0.806113 |

The change of protein expression level was expressed by the ratio of energy/control group. The FC (fold change) ratio >1 (*p* < 0.05) indicates up-regulation and the FC ratio < 1 (*p* < 0.05) indicates down-regulation.

**Supplemental Table 3.** High fiber diet intake up-regulated or down-regulated the mammary gland protein expression in gilts

| **Protein Accession** | **Protein Description** | **Gene Name** | **FC** |
| --- | --- | --- | --- |
| F1RNZ0 | Pleckstrin homology and FYVE domain containing 1 | *PLEKHF1* | 3.879821 |
| F1RKY2 | Serpin family D member 1 | *SERPIND1* | 3.466906 |
| F1SC47 | Delta-1-pyrroline-5-carboxylate synthase | *ALDH18A1* | 2.044517 |
| Q1KS52 | Acid-labile subunit | *ALS* | 2.014179 |
| F1S7J6 | Scaffold attachment factor B | *SAFB* | 1.800233 |
| I3LF89 | Carboxypeptidase N subunit 2 | *CPN2* | 1.747849 |
| A0A287A042 | Uncharacterized protein | *MGAM* | 1.739212 |
| F1RZX9 | Haloacid dehalogenase like hydrolase domain containing 2 | *HDHD2* | 1.697535 |
| R4JU57 | LSm14A protein | *LSm14A* | 1.647118 |
| A0A287BIP6 | Carboxypeptidase Q precursor | *CPQ* | 1.638684 |
| P27917 | Apolipoprotein C-III | *APOC3* | 1.609517 |
| P48819 | Vitronectin | *VTN* | 1.53313 |
| F1S0J2 | Apolipoprotein R precursor | *C4BPA* | 1.51511 |
| A0A287A1B4 | Uncharacterized protein | *PZP* | 1.501272 |
| K7GMF6 | Adhesion G protein-coupled receptor E5 | *ADGRE5* | 1.488158 |
| A0A287A6Q0 | Protein S | *PROS1* | 1.481677 |
| A0SEH3 | Complement component C8 gamma chain precursor | *C8G* | 1.449658 |
| F1SBE5 | Prostacyclin synthase | *PTGIS* | 1.438529 |
| F1RZN7 | Plasma kallikrein | *KLKB1* | 1.432239 |
| A0A287AX82 | Uncharacterized protein |  | 1.417748 |
| F1RK01 | Carboxypeptidase B2 | *CPB2* | 1.40418 |
| O02668 | Inter-alpha-trypsin inhibitor heavy chain H2 | *ITIH2* | 1.331624 |
| F1SIB1 | Prothrombin | *F2* | 1.282599 |
| K7GNZ3 | Uncharacterized protein | *NACA* | 1.247185 |
| F1SN71 | Protein AMBP | *AMBP* | 1.239305 |
| F1SIS9 | NADH dehydrogenase [ubiquinone] 1 alpha subcomplex subunit 10, mitochondrial | *NDUFA10* | 0.380405 |
| F1RKC7 | Phospholipase B-like | *PLBD2* | 0.491439 |
| A0A287BEZ5 | Acetyl-coenzyme A synthetase, cytoplasmic | *ACSS2* | 0.533272 |
| A0A287B9P2 | Scavenger receptor cysteine-rich type 1 protein M130 | *CD163* | 0.657939 |
| I3LNG8 | Stress induced phosphoprotein 1 | *STIP1* | 0.726254 |
| A6XJR1 | Eukaryotic translation initiation factor 4E | *EIF4E* | 0.754958 |

The change of protein expression level was expressed by the ratio of fiber/control group. The FC (fold change) ratio >1 (*p* < 0.05) indicates up-regulation and the FC ratio < 1 (*p* < 0.05) indicates down-regulation.

**Supplemental Table 4.** High-energy and high-fiber diet intake up-regulated or down-regulated the mammary gland protein expression in gilts

| **Protein Accession** | **Protein Description** | **Gene Name** | **FC** |
| --- | --- | --- | --- |
| F1RK90 | Periplakin | *PPL* | 1.871330613 |
| I3LAD5 | Signal transducing adaptor molecule | *STAM* | 1.599270771 |
| F1RKU4 | WD repeat domain 61 | *WDR61* | 1.484981099 |
| A0A286ZVB7 | Dermatopontin | *DPT* | 1.41124428 |
| F1SAT8 | CD93 molecule | *CD93* | 1.27983686 |
| A0A287BAM0 | Dystroglycan | *DAG1* | 1.269908245 |
| F1STV0 | Prolylcarboxypeptidase | *PRCP* | 1.243236538 |
| A0A287B7S0 | CD74 antigen | *CD74* | 1.234020409 |
| Q7YNW7 | MHC class II antigen | *SLA-DRA1* | 1.228925128 |
| A0A287AIQ8 | ATPase H+ transporting V1 subunit E1 | *ATP6V1E1* | 1.201996269 |
| A0A0E3M2Q4 | Transmembrane secretory component poly-Ig receptor (Fragment) |  | 0.370225 |
| A0A286ZQY9 | Ribulose-phosphate 3-epimerase | *RPE* | 0.552541 |
| F1RX84 | Pyridoxal phosphate homeostasis protein | *PLPBP* | 0.663776 |
| I3LPB5 | Creatine kinase B-type | *CKB* | 0.66403 |
| A0A286ZJM4 | DNA replication licensing factor MCM7 | *MCM7* | 0.773441 |
| I3LA84 | Procollagen-lysine,2-oxoglutarate 5-dioxygenase 3 | *PLOD3* | 0.78788 |
| A0A287AFZ8 | Cysteine and histidine-rich domain-containing protein 1 | *CHORDC1* | 0.796851 |
| F1RPU8 | Mitochondrial pyruvate carrier | *MPC2* | 0.801875 |
| A0A287B0W8 | Rho GTPase activating protein 18 | *ARHGAP18* | 0.802324 |
| A0A287AH85 | Adenylate kinase 2, mitochondrial | *AK2* | 0.81694 |

The change of protein expression level was expressed by the ratio of energy and fiber/control group. The FC (fold change) ratio >1 (*p* < 0.05) indicates up-regulation and the FC ratio < 1 (*p* < 0.05) indicates down-regulation.
